# A traveling salesman approach for predicting protein functions

**DOI:** 10.1186/1751-0473-1-3

**Published:** 2006-10-12

**Authors:** Olin Johnson, Jing Liu

**Affiliations:** 1 Department of Computer Science, University of Houston, Houston, US

## Abstract

**Background:**

Protein-protein interaction information can be used to predict unknown protein functions and to help study biological pathways.

**Results:**

Here we present a new approach utilizing the classic Traveling Salesman Problem to study the protein-protein interactions and to predict protein functions in budding yeast *Saccharomyces cerevisiae*. We apply the global optimization tool from combinatorial optimization algorithms to cluster the yeast proteins based on the global protein interaction information. We then use this clustering information to help us predict protein functions. We use our algorithm together with the direct neighbor algorithm [1] on characterized proteins and compare the prediction accuracy of the two methods. We show our algorithm can produce better predictions than the direct neighbor algorithm, which only considers the immediate neighbors of the query protein.

**Conclusion:**

Our method is a promising one to be used as a general tool to predict functions of uncharacterized proteins and a successful sample of using computer science knowledge and algorithms to study biological problems.

## Background

With the development of the genome projects, the focus of research has shifted from studying a single gene or protein to analyzing groups of genes or proteins. In addition to the progress in genome sequencing, researchers have also made great progress in the area called proteomics where people study proteins on the genome level based on their sequence data and their interaction information on a large scale. Protein-protein interactions are very informative for protein function predictions. We speculate that proteins interacting with each other are within the same functional group or within closely related functional groups. By this reasoning, if we have adequate protein-protein interaction information, we can try to predict the functions of uncharacterized proteins based on their interacting neighbors that have been characterized. We can further predict the biological pathways of those proteins. With the rapid progress in identifying protein-protein interactions systematically using two-hybrid experiments and mass spectrometry, we have collected a wealth of information on protein interactions. Here we study in the field of yeast protein clustering and function prediction utilizing a combinatorial optimization tool. The results show that we can cluster the proteins based on their interaction patterns, and we can make predictions of their biological functions based on those clustering. The prediction method works better than the traditional method based only on the direct neighbors of the query protein because our method adopts a global view and hence makes better usage of the information available. With the success in predicting yeast protein functions with the interaction database currently available, we can anticipate continued success as we get more accurate and larger collections of protein-protein interaction information from additional experimental results. Also, we can apply the same methodology to other more complicated organisms, including humans.

In a previously reported study [1], the authors have tried to use graphical display to show protein interaction patterns, to identify protein clusters, and to predict protein functions through visual discerning. This method has given us good intuition about how the proteins are related, but it is not a quantitative or systematic way to study the proteins, especially as the network gets larger.

Other workers in the field have been trying to utilize protein interaction information to predict protein functions using a direct neighbor approach – for a particular protein they try to identify all its interacting neighbors and use a simple mechanism such as voting ("the majority rule") to determine which might be the most likely function [1]. However, many proteins of unknown functions either do not have interacting partners that are characterized or have too few of them for us to trust the voting. This has limited the use of the direct neighbor approach or made its predictions less accurate. In essence the traditional approach adopts a local view of the problem where we only look at the small region of the protein's immediate neighborhood. Here we try to develop an approach that makes use of the global connectivity pattern of the protein interaction network.

The Traveling Salesman Problem (TSP) is a classic problem in the field of graph theory and combinatorial optimization. The Traveling Salesman Problem can be described as following: Given n cities where the distances between any two cities are known, a traveling salesman wants to visit all n cities in a tour so that each city is visited exactly once and the total distance of traveling is minimal [2]. The Traveling Salesman Problem is NP-hard and no polynomial algorithm has been found, but the optimal solutions can be approximated using methods like linear programming or heuristic searching [3]. Among those solutions, the Concorde program [4] is a state-of-the-art program that has provided good quality solutions within reasonable computation time. Concorde is an award winning TSP solver publicly available at .

The Traveling Salesman Problem has many applications in areas such as vehicle routing, job sequencing and data array clustering. The key to convert a data array clustering problem into a Traveling Salesman Problem is to think of the rows and columns as the intermediate cities visited [2,5,6]. In order to make this intuitive notion concrete, we build a target function called the measure of effectiveness (ME) [5] and transform the clustering problem into maximizing this target function, which is a typical global optimization equivalent to TSP. Because of the global and combinatorial nature of the TSP, viewing protein clustering from the TSP perspective automatically makes use of the global information.

## Results

In this approach we download the yeast protein interaction database [7], describe the protein-protein interactions as a connectivity graph represented by the interaction matrix a_ij_, and transform the clustering problem into a Traveling Salesman Problem by using an auxiliary matrix (see methods section.)

**Figure 1:**
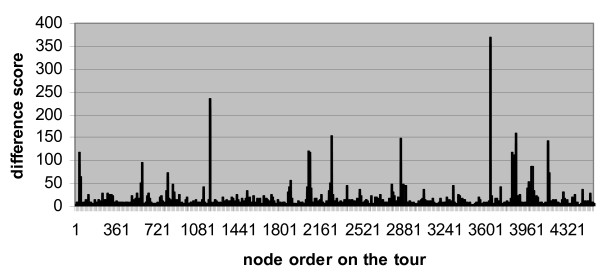
Difference scores between adjacent proteins on the Traveling Salesman Rearrangement of the protein interaction matrix.

**Figure 2:**
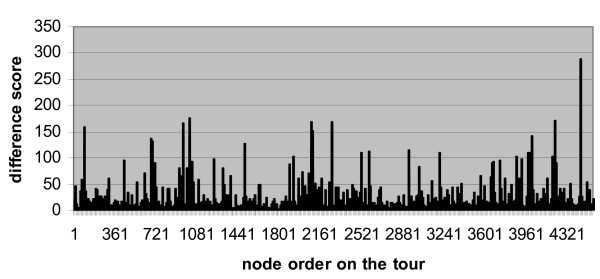
Difference scores between adjacent proteins on a random arrangement of the protein interaction matrix.

When we input the auxiliary matrix to Concorde, we obtain the solution in the form of a permutation of the rows or columns. We re-arrange the rows and columns of the protein interaction matrix according to this permutation and find the permutation produces a matrix with much better patterns of clustering. To quantitatively define the clusters, we compute the difference scores between each two adjacent rows by counting how many corresponding cells are of different values. When we plot the difference scores along the permutated rows we find the scores have a distribution of dozens of peaks over a flat baseline (Figure 1). For comparison, we show in Figure 2 that a random arrangement does not have this pattern.

We analyze the distribution of the difference scores in Figure 1 and use the 95% percentile of the scores as a cut-off to define the boundaries of protein clusters. This way we use a cut-off score of 22 and define 75 clusters for the whole protein network. The cut-off is empirical in that we have tried different cut-off values and have found that a cut-off between 90% and 97% produces good clustering and prediction. When the cut-off is too high, the clusters contain too many proteins some of which are not similar at all and this dilutes the useful information; on the other hand, when the cut-off is too low, the clusters are too small so there is not enough information for protein function prediction.

**Figure 3:**
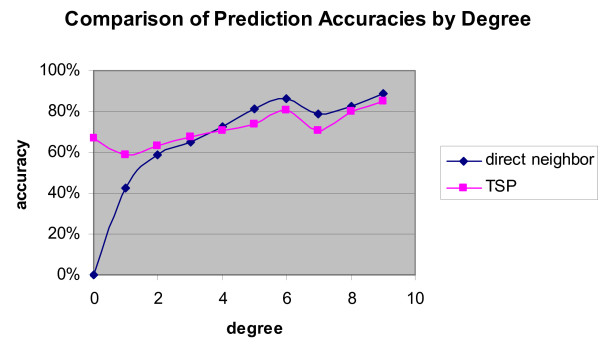
**Prediction accuracy comparison**. Comparison of prediction accuracies between the direct neighbor approach and the new global optimization approach using the TSP algorithms.

We download the protein function catalogue [7]. To get an idea of how the clustering information can contribute to the protein function prediction, we first use the following strategy to predict the functions for a particular protein: for all other proteins in the same cluster, if it is a protein with known functions, we take the vote from that protein and increment the frequency count of each of those functions. We sort the frequency list of the functions and get the top three of the list. We consider the top three our predictions for the most likely functions of the query protein. We use this strategy and the direct neighbor approach [1,8] separately on all known proteins and compare the predictions with the true functions respectively. For each protein with known functions, we compare its true functions with the three predictions we give; if any of the protein's functions belongs to the top three predictions we count that protein as correctly predicted. We look at the prediction accuracy for proteins of different degrees (a protein's degree in the network is defined as the number of its immediate neighbors) and we see the advantage of the global optimization and clustering. The direct neighbor approach cannot predict for a protein with no characterized neighbors because there is no voting input. Plus, if the protein has very few characterized neighbors, the votes are too sparse to give accurate predictions. The Traveling Salesman's approach, on the other hand, can make meaningful predictions in such situations because it allows us to get some useful information from other proteins in the cluster even if the query protein does not have many characterized immediate neighbors. We can see from Figure 3 that when the protein's degree is small, the global prediction method can produce predictions of significantly higher accuracy than the direct neighbor approach. However, when the protein's degree is high, the direct neighbor approach makes slightly better predictions, probably because for such a protein there is already enough information to make good predictions by the direct neighbor approach while the global method introduces extra noise. Figure 3 gives encouraging information because the majority of the proteins have low degrees (About 30% of the proteins have degree of 1). Plus, those lower degree proteins are usually the less characterized ones of the most interest to biologists. Therefore, the advantage our method has on lower degree proteins can be very helpful for predicting biological functions for those proteins.

The analysis above gives us a better understanding of the behaviors of the two approaches. To get the best of both worlds, we use a combination of the two approaches in our prediction. We try various combinations of the two using either the protein's degree or the protein's similarity with the query protein in terms of interaction patterns as the cut-off. We find the following rule simple and effective – when the protein's degree is lower than 4, we use the TSP approach; when the protein's degree is greater than or equal to 4, we use the direct neighbor approach. This way we get a prediction accuracy of 69.72% as compared to the 64.81% from the direct neighbor approach alone.

These results show that our method can give better predictions of protein functions by incorporating the global optimization algorithms. This is especially useful when the protein has no or very few characterized neighbors directly interacting with it. Since we utilize global information of the interactions instead of concerning only the direct interactions in the neighborhood, the method is more robust with regard to local inaccuracies or incompleteness of the information.

## Discussion

One concern of this method is the accuracy of the topology of the network. We need to take into account the false positives and false negatives of the protein-protein interactions obtained from experiments. Incorrect input data affect prediction accuracy and we expect to get better predictions when the input data has better quality.

Another determinant of the method's prediction accuracy is the fineness of the classification. The more coarse-grained the classification, the fewer degrees of freedom there are in the network, and therefore the more likely we will have a correct prediction. On the other hand, to gain more insight from the predictions, we would like the method to be able to predict protein functions on a more fine-grained level.

At this point, we use the first level GO (Gene Ontology) classification for our prediction based on the information we can get from the protein function catalogue. In the future, with more protein annotation and protein-protein interaction data available, we will apply our prediction system on different levels of classification and find a level at which we can predict with meaningful accuracy and the predictions will be most insightful for further biological studies.

Certainly, when we predict the functions for uncharacterized proteins, the ultimate way to validate the predictions is to perform biological experiments. However, the predictions produced by computational methods can give us a good place to begin the experimental exploration and can hence reduce the amount of bench work needed. Instead of speculating wild guesses for an uncharacterized protein's functions, we can form some educated hypotheses and perform experiments to test those hypotheses. The clustering information can also give us some insights into biological pathways because proteins functioning in the same pathway tend to interact with each other and fall into the same cluster. Therefore the global optimization clustering method can help us either to better understand some pathways or to find the missing pieces in them.

The combinatorial optimization tools we use here can easily be used on larger data sets. Given that the Concorde program has solved a 24,978-city TSP problem to the optimum [4], we can expect it to solve the TSP problems obtained from the protein-protein interaction matrices of most organisms. When we get adequate protein-protein interaction information for other organisms, we can use the same methodology to predict protein functions and biological pathways for those organisms.

The reason why our TSP solver based clustering performs better than traditional gene clustering algorithms such as hierarchical clustering or nearest neighbor tree clustering is that the latter methods are essentially greedy bottom-up algorithms where they progressively combine the most similar nodes or clusters at each step till a tree of clusters is built[12]. Greedy algorithms adopt locally best decisions at each step and are likely to face very costly moves at later stages. For this reason, greedy algorithms tend to produce sub-optimal solutions especially for larger problems. In contrast, in our approach, we use Concorde the TSP solver to find the globally optimal solution for the TSP equivalent of our clustering problem. Aiming at global optimization, our method works better especially in the context that there are thousands of nodes (proteins) to be clustered.

Most recently, Climer and Zhang have proposed the TSPCluster algorithm [12], which is an improved TSP-based approach to optimal rearrangement clustering. Their algorithm produces optimal solutions when we have known the number of clusters (k) we are going to cluster the data into and the goal is to find the cluster borders optimally [12]. The rationale is that if we know the number of clusters beforehand, we can use that information, introduce dummy nodes, and modify the object function to minimize the total intra-cluster dissimilarity while tolerating large inter-cluster dissimilarity [13]. Their algorithm works better in situations where we know in advance the range of values for the number of clusters k that are of interest, and we can try a few k values in that range. An example of such situations would be to determine the locations of a few distribution centers based on population clustering [12]. In the more explorative situations like we have now where we do not know how many clusters the proteins are going to be clustered into based on their interaction information, it is better to use our algorithm to globally cluster the proteins and use that clustering information for protein function prediction. After we have performed the study by our algorithm and found out the viable number of clusters k, we can further apply the TSPCluster algorithm with that k value and some nearby values to additionally optimize the clustering.

The success of the method relies on the insight that we need to get information, not only from the protein's immediate neighbors, but also from other components more remotely related. Our method is still a simple one in that we adopt a simple rule where we use the clustering information for proteins with small numbers of neighbors and use direct voting for proteins with more neighbors. If we try to perceive the protein interaction relationship with a more integrated view, we can see that a protein can have direct neighbors, indirect neighbors with a certain number of "bridge" proteins, non-neighbors in the same cluster, and non-neighbors in different clusters. If we assign different weights to those relationships according to the distances of how the proteins are related, and we fine-tune the weights based on our training sets, we hope to get a more sophisticated and more accurate prediction system.

## Conclusion

In this study we have performed yeast protein clustering and function prediction utilizing a combinatorial optimization tool. Our results show that we can cluster the proteins based on their interaction patterns, and that we can make predictions of the biological functions of uncharacterized proteins based on the clustering. The clustering reveals the global patterns of protein-protein interactions within and across functional classes. Although the clustering is not an exact replica of the protein-protein interactions in a proteome, it can be used as a base for protein function prediction. Our prediction method works better than the traditional method based only on the direct neighbors of the query protein in terms of prediction accuracy and prediction robustness with regard to local inaccuracies or incompleteness. The advantage is more prominent when the protein has very few characterized immediate neighbors or no such neighbors at all. The success of our method lies in the fact that it adopts a global view and hence makes better use of the information available.

Our approach is the first one to use the Traveling Salesman Problem, a classical and well studied computer problem and a combinatorial optimization tool, to study the protein-protein interactions from a global point of view. The results show that this approach is a promising one to be used as a general tool to predict functions of uncharacterized proteins. This is a successful sample of using computer science knowledge and algorithms to study biological problems. With the success of being able to predict yeast protein functions more accurately based on the yeast protein database currently available, we can anticipate continued success as we get more complete protein-protein interaction information from additional experimental results. Also, we can apply the same methodology to other more complicated organisms, including humans.

## Methods

### Data and software

We downloaded the yeast protein interaction database and yeast protein function catalogue from the Comprehensive Yeast Genome Database . We downloaded the Concorde TSP solver from Concorde Home . We ran the Concorde program on a Sun ^® ^Ultra 10 work station with a total of 32 GB memory. Our protein function prediction algorithm was implemented in the Perl programming language and was run on an Intel^® ^Xeon processor 2.80 GHz with 2.00 GB RAM installed with Microsoft Windows operating system. We used Microsoft Excel and the SAS^® ^software package for data analysis.

### Transformation of the protein clustering to a Traveling Salesman Problem

Let ρ indicate the permutation of both the rows and the columns because the interaction matrix is a symmetric one. The Measure of Effectiveness (ME) represents the overall similarity and it is the objective function to be maximized. ME is calculated as following [2,9]:

ME(ρ) = 12∑i=1n∑j=1naρ(i)ρ(j)(aρ(i)ρ(j−1)+aρ(i)ρ(j+1)+aρ(i−1)ρ(j)+aρ(i+1)ρ(j))
 MathType@MTEF@5@5@+=feaafiart1ev1aaatCvAUfKttLearuWrP9MDH5MBPbIqV92AaeXatLxBI9gBaebbnrfifHhDYfgasaacH8akY=wiFfYdH8Gipec8Eeeu0xXdbba9frFj0=OqFfea0dXdd9vqai=hGuQ8kuc9pgc9s8qqaq=dirpe0xb9q8qiLsFr0=vr0=vr0dc8meaabaqaciaacaGaaeqabaqabeGadaaakeaadaWcaaqaaiabigdaXaqaaiabikdaYaaadaaeWbqaamaaqahabaGaemyyae2aaSbaaSqaaGGaciab=f8aYjabcIcaOiabdMgaPjabcMcaPiab=f8aYjabcIcaOiabdQgaQjabcMcaPaqabaGccqGGOaakcqWGHbqydaWgaaWcbaGae8xWdiNaeiikaGIaemyAaKMaeiykaKIae8xWdiNaeiikaGIaemOAaOMaeyOeI0IaeGymaeJaeiykaKcabeaakiabgUcaRiabdggaHnaaBaaaleaacqWFbpGCcqGGOaakcqWGPbqAcqGGPaqkcqWFbpGCcqGGOaakcqWGQbGAcqGHRaWkcqaIXaqmcqGGPaqkaeqaaOGaey4kaSIaemyyae2aaSbaaSqaaiab=f8aYjabcIcaOiabdMgaPjabgkHiTiabigdaXiabcMcaPiab=f8aYjabcIcaOiabdQgaQjabcMcaPaqabaGccqGHRaWkcqWGHbqydaWgaaWcbaGae8xWdiNaeiikaGIaemyAaKMaey4kaSIaeGymaeJaeiykaKIae8xWdiNaeiikaGIaemOAaOMaeiykaKcabeaakiabcMcaPaWcbaGaemOAaOMaeyypa0JaeGymaedabaGaemOBa4ganiabggHiLdaaleaacqWGPbqAcqGH9aqpcqaIXaqmaeaacqWGUbGBa0GaeyyeIuoaaaa@7FB1@

With the symmetry between the rows and columns, this function reduces to

ME(ρ) = 2∑i=0n∑j=1najρ(i)ajρ(i+1)
 MathType@MTEF@5@5@+=feaafiart1ev1aaatCvAUfKttLearuWrP9MDH5MBPbIqV92AaeXatLxBI9gBaebbnrfifHhDYfgasaacH8akY=wiFfYdH8Gipec8Eeeu0xXdbba9frFj0=OqFfea0dXdd9vqai=hGuQ8kuc9pgc9s8qqaq=dirpe0xb9q8qiLsFr0=vr0=vr0dc8meaabaqaciaacaGaaeqabaqabeGadaaakeaacqaIYaGmdaaeWbqaamaaqahabaGaemyyae2aaSbaaSqaaiabdQgaQHGaciab=f8aYjabcIcaOiabdMgaPjabcMcaPaqabaaabaGaemOAaOMaeyypa0JaeGymaedabaGaemOBa4ganiabggHiLdaaleaacqWGPbqAcqGH9aqpcqaIWaamaeaacqWGUbGBa0GaeyyeIuoakiabdggaHnaaBaaaleaacqWGQbGAcqWFbpGCcqGGOaakcqWGPbqAcqGHRaWkcqaIXaqmcqGGPaqkaeqaaaaa@4CA3@

Therefore, the network clustering problem becomes a combinatorial optimization problem where the optimal clustering corresponds to the configuration or permutation ρ where ME(ρ) is maximal. This amounts to a Traveling Salesman Problem looking for an optimized permutation ρ with the distance matrix being cij=−∑k=1naikajk
 MathType@MTEF@5@5@+=feaafiart1ev1aaatCvAUfKttLearuWrP9MDH5MBPbIqV92AaeXatLxBI9gBaebbnrfifHhDYfgasaacH8akY=wiFfYdH8Gipec8Eeeu0xXdbba9frFj0=OqFfea0dXdd9vqai=hGuQ8kuc9pgc9s8qqaq=dirpe0xb9q8qiLsFr0=vr0=vr0dc8meaabaqaciaacaGaaeqabaqabeGadaaakeaacqWGJbWydaWgaaWcbaGaemyAaKMaemOAaOgabeaakiabg2da9iabgkHiTmaaqahabaGaemyyae2aaSbaaSqaaiabdMgaPjabdUgaRbqabaGccqWGHbqydaWgaaWcbaGaemOAaOMaem4AaSgabeaaaeaacqWGRbWAcqGH9aqpcqaIXaqmaeaacqWGUbGBa0GaeyyeIuoaaaa@423B@[2,9,10]. We use the formula cij=500−∑k=1naikajk
 MathType@MTEF@5@5@+=feaafiart1ev1aaatCvAUfKttLearuWrP9MDH5MBPbIqV92AaeXatLxBI9gBaebbnrfifHhDYfgasaacH8akY=wiFfYdH8Gipec8Eeeu0xXdbba9frFj0=OqFfea0dXdd9vqai=hGuQ8kuc9pgc9s8qqaq=dirpe0xb9q8qiLsFr0=vr0=vr0dc8meaabaqaciaacaGaaeqabaqabeGadaaakeaacqWGJbWydaWgaaWcbaGaemyAaKMaemOAaOgabeaakiabg2da9iabiwda1iabicdaWiabicdaWiabgkHiTmaaqahabaGaemyyae2aaSbaaSqaaiabdMgaPjabdUgaRbqabaGccqWGHbqydaWgaaWcbaGaemOAaOMaem4AaSgabeaaaeaacqWGRbWAcqGH9aqpcqaIXaqmaeaacqWGUbGBa0GaeyyeIuoaaaa@450F@ to make sure the matrix cells are non-negative numbers. By this analysis, we transform the problem of rearranging the protein-protein interaction matrix into a Traveling Salesman Problem which can be represented by a new matrix C, where cij=500−∑k=1naikajk
 MathType@MTEF@5@5@+=feaafiart1ev1aaatCvAUfKttLearuWrP9MDH5MBPbIqV92AaeXatLxBI9gBaebbnrfifHhDYfgasaacH8akY=wiFfYdH8Gipec8Eeeu0xXdbba9frFj0=OqFfea0dXdd9vqai=hGuQ8kuc9pgc9s8qqaq=dirpe0xb9q8qiLsFr0=vr0=vr0dc8meaabaqaciaacaGaaeqabaqabeGadaaakeaacqWGJbWydaWgaaWcbaGaemyAaKMaemOAaOgabeaakiabg2da9iabiwda1iabicdaWiabicdaWiabgkHiTmaaqahabaGaemyyae2aaSbaaSqaaiabdMgaPjabdUgaRbqabaGccqWGHbqydaWgaaWcbaGaemOAaOMaem4AaSgabeaaaeaacqWGRbWAcqGH9aqpcqaIXaqmaeaacqWGUbGBa0GaeyyeIuoaaaa@450F@. We call this new matrix the auxiliary matrix [11]. The solution to this Traveling Salesman Problem gives us the permutation we need to rearrange the protein interaction matrix.

## Competing interests

The author(s) declare that they have no competing interests.

## Authors' contributions

Olin Johnson conceived of the study, participated in its design and revised the manuscript. Jing Liu carried out the design, implementation and data analysis and drafted the manuscript. Both authors read and approved the final manuscript.
